# Variants of Mitochondrial Genome and Risk of Multiple Sclerosis Development in Russians

**Published:** 2018

**Authors:** M. S. Kozin, O. G. Kulakova, I. S. Kiselev, O. P. Balanovsky, A. N. Boyko, O. O. Favorova

**Affiliations:** Pirogov Russian National Research Medical University, Ostrovitjanova Str. 1, Moscow, 117997, Russia; National Medical Research Center of Cardiology, 3rd Cherepkovskaya Str., 15a, Moscow, 121552 , Russia; Biobank of north Eurasia, Kotlyakovskaya Str., 3, Moscow, 115201, Russia.

**Keywords:** multiple sclerosis, mitochondrial genome, nuclear genome, genetic polymorphism, multilocus analysis

## Abstract

For the first time in the history of ethnic Russians, an association analysis the development of multiple sclerosis (MS) was performed for the mitochondrial haplogroups H, J, K, and U, as well as for the individual mitochondrial DNA (mtDNA) polymorphisms discriminating these haplogroups (m.1719G > A, m. 7028C > T, m.9055G > A, m.10398A > G, m.12308A > G). A total of 283 unrelated patients with the relapsing-remitting form of MS and 290 healthy controls were enrolled in the study. Association of haplogroup J with MS was observed (P = 0.0055, OR = 2.00 [95% CI 1.21–3.41]). After gender stratification, the association remained significant in women (P = 0.0083, OR = 2.20 [95% CI 1.19–4.03]). A multilocus analysis of the association between combinations of mtDNA haplogroups with variants of 38 nuclear immune-related genes and MS risk was carried out. MS–associated biallelic combinations of haplogroup J with the alleles CCL5 rs2107538*A, PVT1 rs2114358*G, TNFSF14 rs1077667*C, and IL4 rs2243250*C, which were not associated with MS individually, were identified. For the combination of haplogroup J and the CCL5*A allele (P = 0.00043, OR = 5.47 [95% CI 1.85–16.15]), a epistatic (synergistic) interaction between the components was established using two statistical criteria: the PFLINT value in the Fisher-like interaction numeric test and the synergy factor, SF (PFLINT = 0.025, SF = 4.32 [95% CI 1.20–15.60]). The combination of haplogroup J and the PVT1*G allele is characterized by PFLINT = 0.084; SF = 3.05 [95% CI 1.00–9.31] and can also be epistatic. Thus, interaction between nuclear and mitochondrial genome components in the risk of developing MS was demonstrated for the first time.

## INTRODUCTION

Multiple sclerosis (MS) is a neurodegenerative disease of the central nervous system; a chronic inflammatory process plays an important role in its pathogenesis. MS typically affects people of working age and, once it has appeared as isolated manifestations of neurological symptoms, it ultimately leads to severe disability [1]. According to the WHO, there are approximately 2.5 million people suffering from MS worldwide. Despite the significant progress achieved in our understanding of the nature of MS and the development of drugs that modify its course, the disease remains among the most socially disrupting conditions.

MS is a disease with a genetic component; the risk of developing it among family members depends on the genetic distance from the proband and reaches its highest values in the closest relatives of the latter [2] but does not obey Mendelian laws. This type of inheritance is typical of polygenic diseases, when there are many independent or interacting polymorphic variants of genes, each of which can only slightly determine disease susceptibility, with the effect often being specific to individual populations (for example, ethnic groups). As a result of many years of research, over 200 independent nuclear loci have been identified using the conventional “candidate gene” approach and modern methods of genome-wide association studies (GWAS). Of these loci, only the region of the major histocompatibility complex class II on chromosome 6 strongly influences the risk of MS, while each of the remaining loci makes a small contribution to susceptibility to MS [3]. However, the combined variability of all identified nuclear loci can explain only approximately 38% of MS inheritance [4].

One of the possible causes underlying this phenomenon, which is called “missing heritability,” may be the unaccounted for effect of mitochondrial genome variability on the risk of developing a polygenic disease. In the case of MS, this assumption agrees well with the data indicating that the disruption of mitochondrial function is one of the key factors leading to neurodegeneration in MS [5]. The main distinguishing features of the mitochondrial genome are known to be only maternal type of inheritance and the absence of recombination. These characteristics allowed researchers to combine different mtDNA variants into haplogroups: groups of related haplotypes present in people who share a common ancestor on the maternal line and inherited one or more nucleotide substitutions. The combination of such substitutions is specific to different haplogroups. In reality, one specific substitution is sufficient for assigning a sample to a haplogroup [6]. Inheritance from one parent leads to a fourfold increase in the effect of genetic drift compared to autosomal markers; as a result, haplogroup frequencies vary greatly in different populations.

To date, there have been approximately 20 studies devoted to the analysis of the association between MS and mitochondrial genome variants: both individual polymorphisms and haplogroups, with the samples being relatively small in some of the cases (see references in review [7]). Among these works, two studies were performed using the GWAS method, and the others involved the “candidate gene” approach. The data presented in these papers are often contradictory, which may be due to the ethnicity of the subjects. In this regard, conducting research on the association of mitochondrial genome variants with the risk of MS in ethnically homogeneous samples is a relevant issue.

The aim of our work is to study the association of the mitochondrial haplogroups H, J, K, and U, which are the most prevalent in European populations [8, 9], and the *MT-RNR2*, *COX1*, *ATP6*, *MT-ND3*, and *MT-TL2 *polymorphisms [10] discriminating these haplogroups against the risk of MS in ethnic Russians. Having taken into account the interaction between the products of mitochondrial and nuclear genes, we also conducted a multilocus analysis of the association of the combinations of mtDNA haplogroups and polymorphic variants of a series of nuclear genes with previously determined frequencies in the sample with the risk of MS and investigated the nature of this effect.

## EXPERIMENTAL

The study included 283 unrelated patients with MS (198 women and 85 men) who were diagnosed with a relapsing-remitting form of MS according to the international McDonald criteria [11]. The mean age of the MS patients at the time of blood collection was 38.0 ± 10.5 years, and the average age of the disease onset was 28.0 ± 9.1 years. All patients underwent treatment at Moscow Multiple Sclerosis Center or Moscow Interregional Department of Multiple Sclerosis at the State Budgetary Health Institution City Clinical Hospital № 24 of the Moscow City Health Department. The control group, which was comparable to the MS group in gender (197 women and 93 men) and age composition (mean age, 40.9 ± 12.9 years), included unrelated healthy individuals. All individuals included in the study were ethnic Russians (according to the survey data, all family members in two generations were Russians) and lived in the European part of Russia. Informed consent to conduct the study was obtained from all individuals. The study was approved by the ethical committee of Pirogov Russian National Research Medical University.

**Genotyping assay**

Total DNA was isolated from blood samples using commercial kits (QIAamp DNA BloodMidiKit).

**Table 1:** Mitochondrial polymorphisms analyzed in the study and used for the determination of haplogroups (H, J, K or U) in individuals

SNP	rs ID	Gene	Gene product	Haplogroup (allele)
m.1719G > A	rs3928305	MT-RNR2	16S ribosomal RNA	I, N1, X2 (1719A)
m.7028C > T	rs2015062	COX1	Cytochrome C oxidase subunit 1 (ETC IV)	H (7028C)
m.9055G > A	rs193303045	ATP6	ATP synthase subunit 6	K (9055A)
m.10398A > G	rs2853826	MT-ND3	NADH dehydrogenase subunit 4 (ETC complex I)	K, J, I (10398G)
m.12308A > G	rs2853498	MT-TL2	Leucine-specific tRNA	U, K (12308G)

Genotyping of single nucleotide polymorphisms (SNP) m.1719G > A, m.7028C > T, m.9055G > A, m.10398A > G, m.12308A > G mtDNA (Table 1) was performed using polymerase chain-reaction-based (PCR) methods. For m.7028C > T, m.10398A > G and m.12308A > G SNPs, restriction fragment length polymorphism (PCR-RFLP) was performed according to the procedure described in [10], with the exception that the DdeI restriction enzyme was replaced with its isoschizomer, BstDEI. Polymorphism m.9055G > A was genotyped by PCR-RFLP using the primers 5’-TTAAGGCGACAGCGATTTCT-3’, 5’-TACTGCAGGCCACCTACTCA-3’ and the AspLEI restriction enzyme. Polymorphism m.1719G > A was genotyped by real-time PCR. Amplification of the studied region was carried out using the primers 5’-GCTAAACCTAGCCCCAAACC-3’ and 5’-GCGCCAGGTTTCAATTTCTA-3’. SNP analysis was conducted using probes specific to the A (5’ HEX-CCTTACTACCAGACAACCTTAAC-CAAACC-3’BHQ1) and G (5’ FAM-CCTTACTACCAGACAACCTTAGCCAAACC-3’BHQ1) alleles.

**Mitochondrial haplogroup **(**H, J, K or U)**

was determined based on the combination of the marker SNPs presented in *Table 1*, according to [10]. Haplogroup H was defined as the extended haplotype G1719, C7028, G9055, A10398, A12308; haplotype G1719, T7028, G9055, G10398, A12308 was identified as haplogroup J; haplogroups K and U were defined as haplotype G1719, T7028, A9055, G10398, G12308 and haplotype G1719, T7028, G9055, A10398, G12308, respectively.

**Statistical analysis**

The search for individual mitochondrial SNPs and the mitochondrial haplogroups associated with MS, as well as combinations of haplogroups with the carriage of alleles/genotypes of a series of nuclear genes, which had been previously identified (unpublished data), was conducted using the APSampler software [12], based on Monte Carlo Markov chains and Bayesian nonparametric statistics [13]. The significance level of the identified associations was assessed using the validation tools included in the APSampler software and based on Fisher’s exact test, evaluation of the corresponding odds ratio (OR), and a 95% confidence interval (CI). Associations were considered significant if the P value was less than 0.05, provided that the 95% CI of the OR did not cross 1.

Possible nonlinear interaction (epistasis) between alleles in the identified biallelic combinations was revealed using a previously proposed approach [14]. The method is based on the assessment of the nature of an interaction between the alleles (or genotypes) of two loci in their combined carriership using the two previously described statistical criteria: P_FLINT_ value of the exact three-way Fisher-like interaction numeric test (F_LINT_) [15] and based on the synergy factor (SF) values and 95% CI [16]. SF, P_FLINT_, and 95% CI were assessed using tools included in the APSampler software. Interaction for biallelic combinations was considered epistatic for P_FLINT_ lower than 0.05, provided that the 95% CI of the SF did not cross 1.

## RESULTS

Analysis of the frequencies of the mitochondrial genome variants m.1719G > A, m.7028C > T, m.9055G > A, m.10398A > G, and m.12308A > G was performed in MS patients and control individuals belonging to the Russian ethnic group. There were no significant differences in SNP frequencies when comparing the total samples of patients with MS and control individuals, nor when comparing patients with healthy men and healthy women separately (data not shown).

**Fig. 1:**
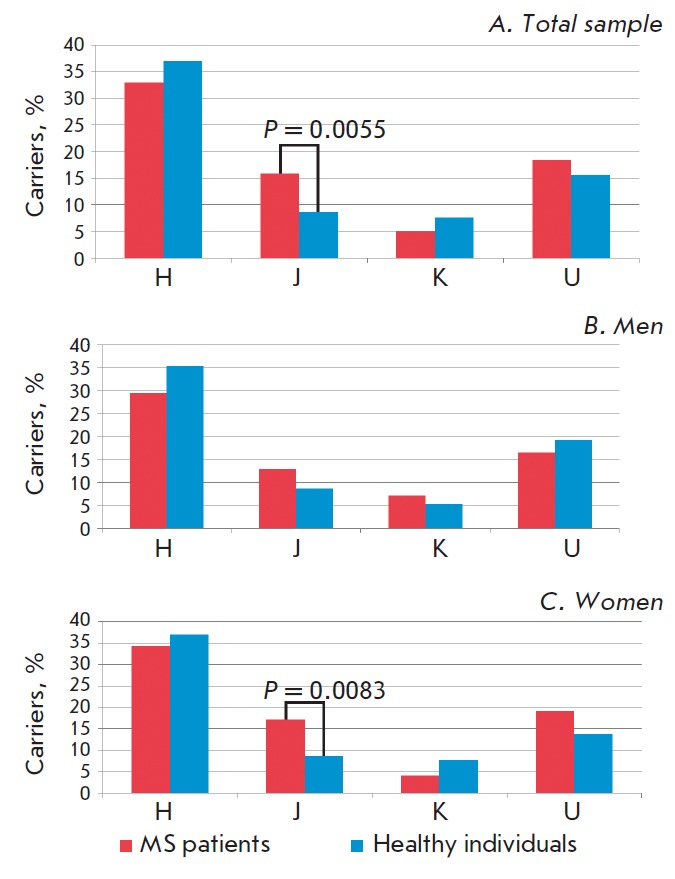
The frequencies of haplogroups H, J, K, and U in MS patients and healthy individuals. A – total sample (283 patients with MS, 290 healthy individuals); B – men (85 patients with MS, 93 healthy individuals); C – women (198 patients with MS, 197 healthy individuals)

Mitochondrial haplogroup (H, J, K or U) was determined by genotyping of the above-indicated marker SNPs based on their combinations. The frequency of haplogroup J in patients with MS (15.9%) is almost 2 times higher than the frequency in the control group (8.6%) and significantly associated with the risk of MS (P = 0.0055; OR = 2.00 [95% CI 1.21–3.41]). No association with MS was found for haplogroups H, K, and U (*Fig. 1A*).

Due to the fact that MS is significantly more common in women than in men and the presence of gender differences in genetic risk factors for the disease [17], the association analysis of haplogroups H, J, K, U with MS was also performed separately for men and women. No significant associations with any of the studied haplogroups were found in men (*Fig. 1B*). On the contrary, association of haplogroup J with MS was revealed both in women and the total sample (P = 0.0083; OR = 2.20 [95% CI 1.19–4.03]) (*Fig. 1B*).

Current evidence suggests that mitochondria functioning is altered in chronic neuroinflammation specific to MS [7]. In order to assess the possible interaction of mitochondrial and nuclear genes, we carried out a multilocus analysis of the association of the carriership of combinations of each of the studied mtDNA haplogroups with polymorphic variants of the 37 nuclear genes involved in the immune system functioning with MS using the APSampler software.

The studied genes include the major histocompatibility complex HLA-*DRB1 *gene, the key gene determining susceptibility to MS, as well as *CD58, VCAM1, EVI5, EOMES, CD86, IL7RA, TCF7, IL22RA2, IRF5, PVT1, IL2RA, CD6, CXCR5, TNFRSF1A, CLEC16A, IRF8, STAT3, TYK2, TNFSF14*, and *CD4*, association with which MS has been shown using GWAS. These genes fall under the following criteria: at least two independent GWAS demonstrated association with MS; at the same time, a whole genome level of significance was reached (*P *≤ 5 × 10-8) in at least one study, while other studies showed a *P *value not exceeding 1 × 10-5 [18]. Of particular interest to us was *CLEC16A*, which is located in a relatively gene-rich region of chromosome 16 containing three linkage blocks. This area also includes *SOCS1*, one of the most important regulators of cytokine expression [19]. For this reason, we included two polymorphic sites located in the adjacent intergenic region of the chromosome, *CLEC16A-SOCS1 *(rs1640923) and *SOCS1-TNP2 *(rs243324), in the analysis. Products of the remaining genes under study are involved in the process of inflammation and/ or described as associated with various autoimmune diseases, including MS. They include genes encoding the components of the cytokine/chemokine system, *IL4, IL6, IL17A, IFNB1, IFNG, TNF, TGFB1, CCL5, IFNAR, IFNAR2, CCR5, *as well as genes the products of which participate in the regulation of T lymphocyte activity: namely, the costimulatory molecule *CTLA4 *and immunoproteasome subunit *PSMB9 *required for processing of peptides prior to their presentation in MHC class I. Glypican 5 gene (*GPC5) *has been included in the study since its polymorphisms are known to be associated with the nature of the response of MS patients to immunomodulatory therapy with interferon-β [20]. Frequency of a minor allele was at least 0.05 for all studied polymorphic sites. Carrier frequencies of alleles and genotypes of nuclear genes in the analyzed sample had been determined by us earlier.

**Table 2:** Association of combinations between mitochondrial haplogroup J and carriage of alleles/genotypes of nuclear genes with MS (according to the results of a multilocus analysis)

Haplogroup, allele or genotype	Number of carriers, %		P	OR MS patients [95% CI]
	MS patients [95% CI] (N = 283)	Healthy donors (N = 290)		
Distinct genetic variants				
^#^Haplogroup J	45 (15.9)	25 (8.6)	0.0055	2.00[1.19–3.37]
CCL5 rs2107538*A	110 (38.8)	105 (36.2)	0.44	1.04[0.74–1.46]
PVT1 rs2114358*G	169 (59.7)	170 (58.6)	0.43	1.04[0.75–1.46]
TNFSF14 rs1077667*C	266 (93.9)	261 (90.0)	0.064	1.72[0.90–3.27]
IL4 rs2243250*C	267 (94.3)	264 (91.0)	0.14	1.52[0.79–2.92]
^#^CLEC16A-SOCS1 rs1640923*A/A	221 (78.0)	203 (70.0)	0.020	1.51[1.03–2.20]
Combinations of genetic variants				
^#^Haplogroup J + CCL5*A	21 (7.4)	4 (1.4)	0.00043	5.47[1.85–16.15]
^#^Haplogroup J + PVT1*G	35 (12.4)	14 (4.8)	0.00093	2.78[1.46–5.29]
^#^Haplogroup J + TNFSF14*C	44 (15.5)	21 (7.2)	0.0013	2.35[1.35–4.07]
^#^Haplogroup J + IL4*C	44(15.5)	21 (7.2)	0.0013	2.35[1.35–4.07]
^#^Haplogroup J + CLEC16A-SOCS1*A/A	39 (13.7)	17 (5.9)	0.0011	2.56[1.41–4.63]

^#^ - Significant associations

Combinations with the alleles of the nuclear genome significantly associated with the risk of MS were found only for haplogroup J (*Table 2*). As a second component, these biallelic combinations included the alleles *CCL5 *rs2107538*A, *PVT1 *rs2114358*G, *TNFSF14 *rs1077667*C and *IL4 *rs2243250*C, which individually were not significantly associated with MS, and genotype *CLEC16A-SOCS1 *rs1640923*A/A, which was significantly associated with MS (*P *= 0.020 and OR = 1.51 [95% CI 1.03–2.20]). All combinations were characterized by a high level of significance (*P *in the range of 0.00043 to 0.0011) exceeding the significance of association with MS for haplogroup J at least 5 times. At the same time, an increase in the OR was observed: OR was equal to 5.47 for the most significant combination (haplogroup J + CCL5*A), which exceeds the OR showed for haplogroup J almost 3 times.

**Table 3:** Analysis of the nature of interactions between the components of combinations: carriership of mitochondrial haplogroup J and alleles/genotypes of nuclear genes

Combination of genetic variants	P_FLINT_	SF [95% CI]
Haplogroup J + CCL5*A	0.025^#^	4.32[1.20–15.60]^#^
Haplogroup J + PVT1*G	0.084	3.05[1.00–9.31]^#^
Haplogroup J + TNFSF14*C	0.31	4.25[0.38–47.60]
Haplogroup J + IL4*C	0.14	6.85[0.65–72.30]
Haplogroup J + CLEC16ASOCS1*A/A	0.34	2.24[0.63–7.97]

^#^ - Significance criteria

Increase in the significance level for the association with MS that is observed for combined carriership of haplogroup J and the alleles (or genotypes) of nuclear genes may occur as a result of summing up their mutually independent contributions or as a result of their positive epistatic (synergistic) interaction. In order to assess whether such interactions take place in the case of the identified combinations, we determined their SF and P_FLINT_ values. For the combination of haplogroup J with the allele rs2107538*A of CCL5, P_FLINT_ equals 0.025 and SF is equal to 4.32 [95% CI = 1.2–15.6] (*Table 3*). Thus, it has been demonstrated that the increase in the risk of MS observed in combined carriership of haplogroup J with the CCL5*A allele is associated with a synergistic epistatic interaction between these genetic variants. Combination of haplogroup J with PVT1*G is characterized by SF = 3.05 with CI not crossing 1. According to this criterion, it falls under the definition of epistatic interaction. However, the P_FLINT_ value (0.084) does not reach the level of significance and we cannot state that this combination is epistatic. SF values for 95% CI and P_FLINT_ obtained for the remaining combinations were shown to be not significant.

## DISCUSSION

MS is a clinically and genetically heterogeneous disease [21]. For this reason, sampling criteria are of great importance for obtaining reliable results. We can state that the studied group of patients was fairly representative. All patients were diagnosed with the most common relapsing-remitting form of MS, which is characterized by periods of exacerbation and remission. The ratio of MS women and men and the average age of MS onset were close to that described in [22]. Gender ratio and the age of individuals in the control group did not differ significantly from those in the group of patients. Frequencies of the mitochondrial haplogroups in the control group were close to the frequencies determined earlier for the European part of Russia [8, 9].

This paper presents the first analysis of an association of MS with mitochondrial SNPs (m.1719G > A, m.7028C > T, m.9055G > A, m.10398A > G, m.12308A > G) and mtDNA haplogroups (H, J, K, U) in ethnic Russians. Of the SNPs included in the study, association of m.1719G > A, m.10398A > G, and m.9055G > A SNPs with MS was analyzed in three European populations (Hispanics, Norwegians, Germans); no significant association with MS was observed for any of these populations [23], which is consistent with our results. However, SNP m.9055G> A (haplogroup K) showed a significant association with the disease in caucasian Americans [24], which probably reflects their genetic differences from Europeans.

A significant association of haplogroup J with MS found in our study had been previously shown for some European ethnic groups [23, 25-27] (but not for all of the studied individuals), as well as for Americans of European descent [28] and Persians from Iran [29]. Thus, we replicated the previously obtained data on the association of haplogroup J with the risk of MS in ethnic Russians. When stratifying our sample by gender, the association of haplogroup J with MS remained significant in women, but not in men, with the level of significance being lower in women than in the sample that was not divided by gender. It is possible that these results are due to the insufficient number of men in the sample. Previously published data on the relationship between haplogroup K and MS in the American [24] and Persian [30] populations were not reproduced for the Russian population in our study.

Increased risk of MS in individuals carrying haplogroup J is probably due to its specific impact on the functioning of mitochondria and cells in general. Indeed, the studies carried out using “cybrids,” i.e. cells with an identical nuclear genome but different mitochondria, have showed that it is the carriage of haplogroup J that leads to significant changes in the cells. For instance, it was shown [31] that the global level of DNA methylation in the peripheral blood cells of haplogroup J carriers is higher than that for carriers of other haplogroups; it is also higher in cybrids containing this variant of mtDNA (J cybrids) compared to other cybrids. At the same time, ATP concentration and production of free radicals were shown to be reduced in J cybrids [31]. Polymorphism m.295C > T of the mtDNA control region (one of the SNPs determining haplogroup J) was shown to affect the processes of mtDNA transcription and replication. In particular, if the T allele is carried, binding of the mitochondrial transcription factor A (TFAM) to mtDNA is enhanced and the content of mtDNA in J cybrids becomes two time higher in comparison with H cybrids [32]. Unfortunately, the authors do not present data on microscopic examination of cells, and, therefore, it is unclear which of the previously described phenomena determines the increase in the amount of mtDNA: an increase in the number of mitochondria or an increase in the mtDNA copy number per mitochondria. However, one can assume that increase in the mtDNA content in carriers of haplogroup J is a compensatory response to a decrease in ATP production. One of the key features of MS is the increase in energy consumption for maintaining structural integrity and functioning of axons at the sites of demyelination, which can be compensated at the initial stages by an increase in the number of mitochondria and the size of stationary mitochondria, as well as by an increase in the speed of axonal mitochondrial transport [33]. One can assume that the carrier of haplogroup J has already run out of the compensatory reserve of neurons by the time of disease manifestation.

Using a multilocus analysis, we have demonstrated the involvement of a number of combinations of haplogroup J with the alleles CCL5, PVT1, TNFSF14, and IL4 that are not individually associated with MS in the development of MS; these combinations are characterized by a greater significance of the association with the disease than haplogroup J only. Regardless of whether the observed cumulative effect occurs because of the summing up of the independent contributions of the two components of each of the combinations or due to the epistatic interactions between them [34], the obtained results allow us to suggest that not only the genes identified in combinations with haplogroup J, but also nuclear genes are involved in the formation of susceptibility to MS.

The products of the protein-encoding genes *CCL5*, *TNFSF14, *and *IL4*, which have been studied in combination with haplogroup J, share a similar role and participate in the functioning of the cytokine/ chemokine system. CCL5 is a chemokine that acts as a chemoattractant of monocytes, memory T cells, and eosinophils. An increase in the concentration of CCL5 in the cerebrospinal fluid can serve as one of the markers of MS progression [35]. Proinflammatory cytokine TNFSF14, the fourteenth member of the superfamily of tumor necrosis factors, can function as a co-stimulator in lymphocyte cell activation, can stimulate T cell proliferation, and induce apoptosis of some types of tumor cells. IL4 is one of the key cytokines regulating differentiation of naive (Th0) T helpers into Th2 cells and differentiation of B cells into plasma cells. A multilocus analysis of mitochondrial and nuclear genome variants allowed us to replicate the data that were previously obtained for other populations on association of rs2107538 of the gene *CCL5 *[36], rs1077667 of the gene *TNFSF14 *[37], and rs2243250 of the gene *IL4 *[38, 39] with the risk of MS in ethnic Russians.

Another gene that has been identified by us as a component in the combination of haplogroup J with MS, namely, *PVT1*, encodes long non-coding RNA presumably involved in cell cycle regulation [40] and contains a cluster of six miRNA genes [41]. The SNP rs2114358 included in our study is located in intron 5 of the gene *PVT1*, which also encodes miR-1206, and, as shown by *in silico *analysis, affects the structure of mature miR-1206 [42]. The GWAS method revealed association of MS with another polymorphism in *PVT1*, rs4410871 [37], which, like rs2114358, is part of the miRNA gene (*MIR1204*, located in intron 1 of the gene *PVT1*).

We have established the fact of synergistic interaction between carriage of haplogroup J and the allele rs2107538*A of the gene *CCL5*. Elucidation of the molecular mechanism of this interaction is a challenge for the future. However, chemokine CCL5 is known to play an essential role in the metabolism of glutamic acid in the central nervous system by modulating glutamatergic signal transduction [43], while the synthesis of glutamate occurs with direct involvement of mitochondrial enzymes [44]. Moreover, it was found that glutamate homeostasis is disturbed at the sites of damage in MS [45]. Moreover, glutamate excitotoxicity, which develops in this case, is one of the mechanisms of neuronal damage [46]. These processes may underlie the observed synergistic effect of the combination of haplogroup J and allele CCL5*A on the development of MS. Another biallelic combination that has been shown in the current study to be associated with the risk of MS, which includes haplogroup J and allele rs2114358*G of the gene *PVT1*, meets only one of the two criteria for nonlinear interaction between genetic variants. We would like to suggest that expanding the sample size will allow us to prove the synergistic nature of this combination.

Thus, we obtained data indicating epistatic interaction between haplogroup J and the gene *CCL5 *and, apparently, the gene *PVT1*. Thus, interaction of the components of the nuclear and mitochondrial genomes in the formation of a risk of MS has been demonstrated for the first time. The obtained results certainly require reproduction on an independent sample.

## Abbreviations

FLINT: Fisher-like interaction numeric test
GWAS: genome-wide association study
SF: synergy factor
SNP: single nucleotide polymorphism
ATP: adenosine triphosphate
CI: confidence interval
mtDNA: mitochondrial DNA
NAD: nicotinamide adenine dinucleotide
OR: odds ratio
RFLP: restriction fragment length polymorphism
PCR: polymerase chain reaction
ETC: electron transport chain
